# Machine learning driven reservoir property modeling of the AEB-3E reservoir in the Berenice field Egypt

**DOI:** 10.1038/s41598-026-53328-3

**Published:** 2026-05-25

**Authors:** Amr M. Eid, Walid M. Mabrouk, Mohammed Amer, Ahmed Metwally

**Affiliations:** Geophysics Department, Faculty of Science, Cairo University, Giza, 12613 Egypt

**Keywords:** Reservoir characterization, EMBER machine learning, Property modeling, AEB-3E reservoir and stochastic modeling, Energy science and technology, Engineering, Solid Earth sciences

## Abstract

Accurate reservoir characterization in structurally complex fields is essential for optimizing hydrocarbon exploration and production. This study presents a detailed analysis of the AEB-3E reservoir within the Berenice Oil Field. It integrates well log and 3D seismic data using the EMBER (Ensemble Machine Learning for Better Estimation of Reservoir properties) workflow. Four wells provided composite logs, including gamma ray, density, neutron porosity, resistivity, and sonic measurements. These logs were quality-controlled, depth-matched, corrected, and upscaled to a 3D geological grid. Seismic-derived attributes were used to capture lateral heterogeneity and structural trends. They also enabled improved interwell property prediction in areas with sparse data. Petrophysical properties (shale volume, effective porosity, and water saturation) were modeled using EMBER. The method combines ensemble decision tree regression with embedded geostatistical features. This allows spatial continuity and stratigraphic relationships to be honored. Deterministic results indicate that the AEB-3E interval is predominantly clean. Average shale volume ranges from 0.11 to 0.18, effective porosity from 0.11 to 0.147, and water saturation from 0.28 to 0.45. Cross-sectional analysis from eastern and western parts of the field confirms lateral consistency. It also highlights localized variations controlled by structural features. Stochastic simulations were used to quantify uncertainty. They reveal low to moderate variability and indicate higher uncertainty near faults. The EMBER workflow significantly reduces modeling time and manual effort. At the same time, it delivers reliable and interpretable reservoir property predictions. Despite the limitation of only four wells, the methodology shows potential for application in similar geological settings. Overall, integrating machine learning with seismic and well data shows promise as an effective framework for reservoir evaluation, offering valuable insights to support informed decision-making and risk assessment in hydrocarbon development. However, the limited dataset warrants cautious interpretation, and further validation is recommended for broader application.

## Introduction

 Despite significant advances in geostatistical and data-driven reservoir modeling, accurate prediction of petrophysical properties in heterogeneous clastic systems remains challenging. Classical geostatistical approaches, including variogram-based kriging and sequential simulation techniques, provide a robust statistical framework for spatial interpolation and uncertainty quantification but are fundamentally limited by assumptions of stationarity and predefined spatial continuity structures^1–3^. Although extended stochastic and hybrid geostatistical workflows have improved the representation of reservoir heterogeneity and uncertainty in field-scale applications^2,4^, they still rely on explicit spatial parameterization and may not fully capture non-linear relationships between seismic attributes and petrophysical properties in structurally complex reservoirs. Recent developments in machine learning-based reservoir characterization have demonstrated improved capability in integrating multi-source datasets and capturing non-linear trends without strict statistical assumptions^3^ yet many of these approaches remain sensitive to feature selection procedures, parameter tuning, and reproducibility constraints. These limitations are particularly relevant in the Lower Cretaceous Alam El Bueib Formation, where strong facies variability and fault-controlled compartmentalization govern reservoir heterogeneity and fluid distribution, making conventional deterministic or semi-deterministic approaches insufficient for reliable spatial prediction. Accordingly, there remains a need for a unified and reproducible framework that integrates seismic, well-log, and structural data while preserving geological consistency and explicitly quantifying uncertainty^1,2,4–7^.

This study addresses this gap by applying the EMBER ensemble-based machine learning workflow to the AEB-3E reservoir in the Berenice Field, enabling non-linear property prediction and stochastic uncertainty characterization within a reproducible geostatistical–machine learning framework.

EMBER combines data-driven learning with classical geostatistical concepts to generate reliable reservoir property predictions, while providing an estimate of the associated uncertainties within the available dataset. Unlike traditional geostatistical approaches, EMBER does not require strict assumptions of stationarity or extensive preconditioning of input data, making it particularly suitable for complex and heterogeneous reservoirs^8,9^.

The EMBER algorithm is fundamentally based on ensemble learning techniques using regressive decision trees, which are trained by integrating multiple sources of information, including upscaled well logs, seismic-derived attributes, facies models, trends, and geometric grid properties^8,10^. A distinctive feature of EMBER is the embedding of geostatistical prediction models, such as kriging, directly into the machine learning framework to account for stratigraphic and spatial continuity. This hybrid approach allows EMBER to honor spatial patterns and geological constraints while benefiting from the predictive power of machine learning, resulting in data-driven property models with improved spatial conformance and realism.

In addition to deterministic property estimation, EMBER enables stochastic simulations by constructing a conditional probability distribution at each grid cell^11^. From these local distributions, multiple realizations can be generated to represent reservoir variability and heterogeneity. The workflow provides several quantitative uncertainty outputs, including estimation mean, median, spread (P90–P10), probability volumes, and user-defined quantiles. Such outputs are critical for identifying sweet spots, assessing reservoir risk, and constraining dynamic flow simulations, thereby supporting more informed decision-making in reservoir development and management^10,12^.

Furthermore, EMBER offers advanced controls such as blind well testing for model validation and simulation intervention mechanisms that allow results to be biased toward selected properties or target means while remaining constrained within the predicted uncertainty envelope. This ensures that simulated models remain geologically plausible and statistically consistent with available data. Overall, EMBER provides a flexible and powerful framework for integrating machine learning and geostatistics within Petrel, enabling reliable petrophysical property modeling and uncertainty quantification in both data-rich and data-scarce reservoir settings^9^.

**Fig. 1:**
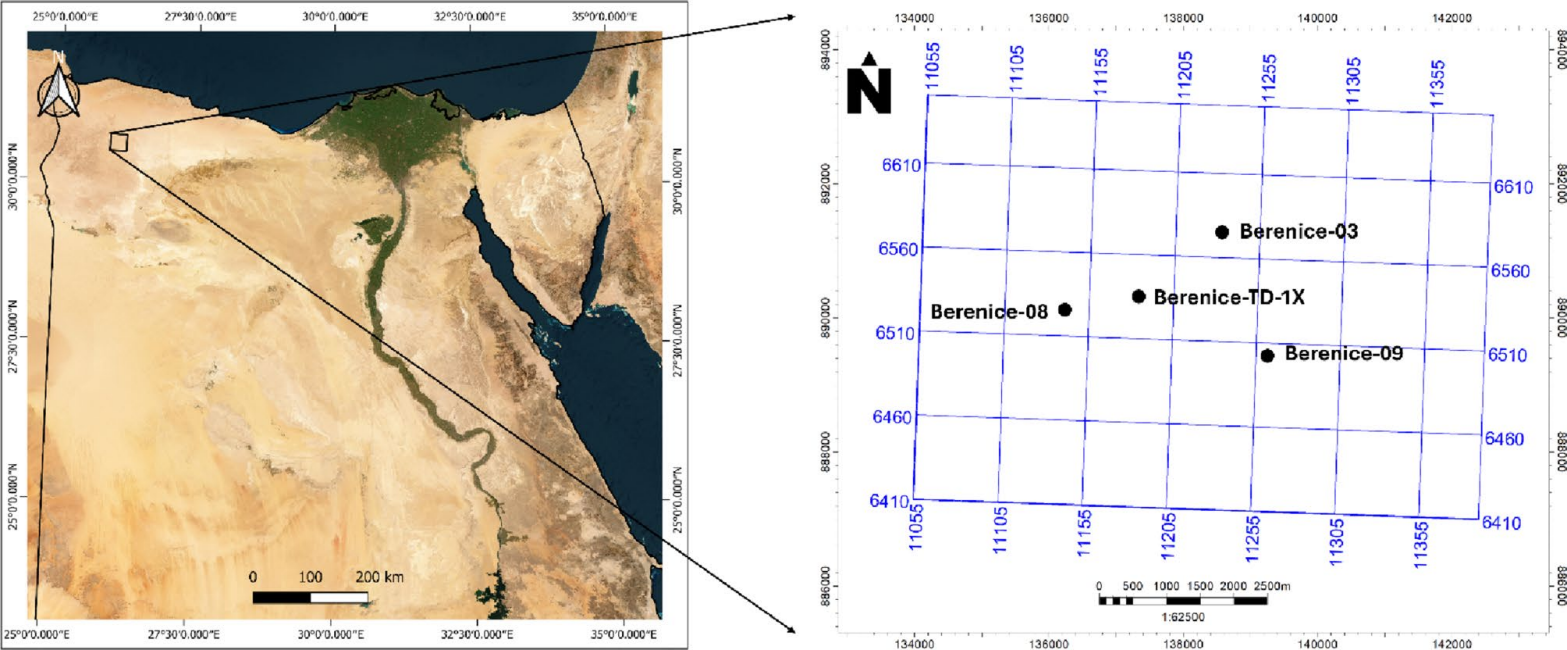
Geographical representation of the Berenice Field showing the coverage of the 3D seismic dataset and the spatial arrangement of the investigated wells within the study area.

The primary aim of this manuscript is to demonstrate the value and applicability of the EMBER machine learning–based property modeling workflow for improving reservoir characterization in geologically complex and data-limited settings, using the Berenice oil field in the Western Desert of Egypt as a representative case study (Fig. 1). The Berenice Field is characterized by significant stratigraphic and structural heterogeneity, variable reservoir quality, and uneven well spacing, conditions that are typical of many producing fields across the Western Desert^13–15^. By integrating well log data with seismic-derived attributes and geological constraints through an ensemble machine learning framework, EMBER enables more reliable petrophysical property prediction and uncertainty quantification, particularly in interwell regions. The outcome of this study is intended to establish Berenice as an effective analogue field, demonstrating how EMBER-based workflows can be transferred and applied to other oil and gas fields in the Western Desert to reduce modeling uncertainty, enhance reservoir understanding, and support more informed development and management decisions.

## Geological setting

The Berenice Field is located in the northeastern part of the Faghur Basin, Egypt’s Western Desert, and is characterized by a structurally complex regime dominated by faulted tilted blocks and segmented structures that control reservoir compartmentalization and continuity^16,17^.

**Fig. 2:**
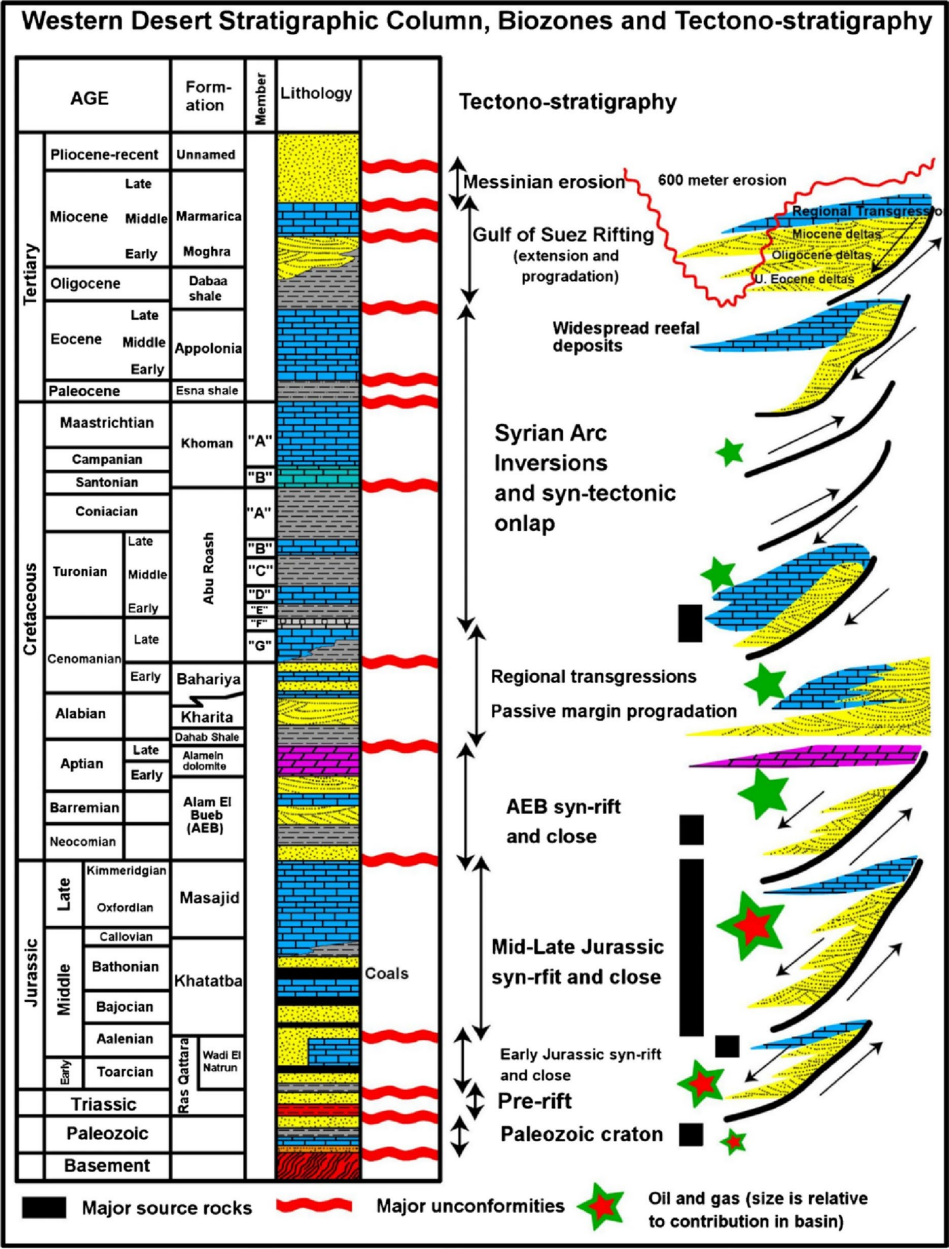
Stratigraphic succession of the Northwestern Desert depicting the principal lithological units and their stratigraphic hierarchy as a regional reference for the study^16,23^.

The studied interval belongs to the Lower Cretaceous Alam El Bueib Formation, a major hydrocarbon-bearing unit in the Western Desert (Fig. 2). Within this formation, the AEB-3E sub-member represents the primary reservoir target due to its relatively consistent development and reservoir quality^14,15,18^.

Lithologically, the AEB-3E consists of interbedded fine- to medium-grained sandstones, siltstones, and shales deposited in fluvial to shallow marine environments. This depositional setting results in significant vertical and lateral heterogeneity, expressed as variations in shale volume, effective porosity, and water saturation^15,19^.

The combined effects of depositional facies variability and fault-controlled segmentation led to strong spatial heterogeneity in reservoir properties and fluid distribution. Faulting further enhances compartmentalization, resulting in variable petrophysical responses across structural blocks^20–22^.

## Material and methodology

### Data description

This study integrates well log and seismic data to characterize the AEB-3E reservoir within the Berenice Oil Field (Fig. 1). The dataset comprises four wells distributed across the field, each containing a complete suite of composite well logs used for petrophysical evaluation and machine learning training. The available logs include gamma ray, density, neutron porosity, resistivity, and sonic measurements, which were quality-controlled, depth-matched, and environmentally corrected prior to analysis. These logs were subsequently upscaled to the 3D geological grid to ensure consistency between well-scale measurements and grid-based modeling.

In addition to well data, a 3D seismic survey covering the entire Berenice Field was utilized to provide spatially continuous information between wells. Seismic-derived attributes were extracted from the interpreted seismic volume and used as secondary input features to capture lateral heterogeneity and structural trends within the reservoir. The integration of seismic attributes with well log data improves interwell property prediction, particularly in structurally complex and data-sparse areas. A summary of the available wells, well logs, and seismic attributes used in this study is provided in Table-1.

**Table 1:** Summary of available wells, well logs, and seismic attributes used in this study.

Category	Details
Wells	4 wells distributed across the Berenice Field
Well log types	Gamma Ray, Density, Neutron Porosity, Resistivity, Sonic
Well log processing	Quality control, environmental corrections, upscaling to 3D grid
Target properties	Shale Volume (Vsh), Effective Porosity (Φe), Water Saturation (Sw)
Seismic data	3D seismic survey covering the entire field
Seismic attributes	Variance, RMS amplitude, Chaos, Seismic Edge, Relative Acoustic Impedance, Second Derivative

### Petrophysical property preparation

Petrophysical properties, including shale volume (Vsh), effective porosity (Φe), and water saturation (Sw), were computed from the composite logs using standard industry workflows. Shale volume was derived primarily from gamma ray responses, while effective porosity was calculated from density–neutron relationships after shale correction. Water saturation was estimated using resistivity-based models calibrated to reservoir lithology and fluid properties. The calculated properties were then upscaled to the reservoir grid and used as target variables for machine learning–based property modeling^15,24–28^. 

### EMBER machine learning workflow

Property modeling was carried out using the EMBER algorithm implemented in *Petrel-2024* (SLB), which integrates ensemble-based regression trees with embedded geostatistical conditioning to predict reservoir properties within a 3D geological framework. The workflow was designed as a fully traceable and reproducible sequence, with explicit controls to minimize interpretational bias, prevent data leakage, and ensure that uncertainty reflects geological variability rather than algorithmic artifacts.

The workflow began with data preparation and deterministic upscaling of well-log-derived petrophysical properties, including shale volume (Vsh), effective porosity (Φe), and water saturation (Sw). These properties were computed using standard petrophysical interpretation and then consistently upscaled to the simulation grid prior to any model training. This step ensured strict alignment between well data and grid representation and avoided any form of sampling inconsistency or post hoc adjustment that could introduce bias.

Following upscaling, a fixed and pre-defined feature set was constructed to serve as model inputs. This feature space included seismic attributes such as variance, RMS amplitude, chaos, seismic edge, relative acoustic impedance, sweetness, and second derivative, all computed from a single seismic dataset using identical processing parameters to guarantee consistency. In addition, structural coordinates (X, Y, Z) and grid indices (I, J, K) were incorporated to capture spatial trends and stratigraphic organization. The workflow also included internally generated geostatistical predictors derived from kriging-based estimates embedded within Petrel-2024. Importantly, the feature set was frozen prior to training, ensuring that no iterative modification feature engineering was performed, thereby eliminating feature-selection bias.

Rather than applying manual dimensionality reduction or subjective filtering, feature relevance was assessed exclusively through EMBER’s internal quality-control diagnostics. These diagnostics compute variable importance based on each predictor’s contribution to error reduction across the ensemble of regression trees. This design ensures that feature evaluation remains fully data-driven and does not impose prior geological assumptions on variable selection. Correlated seismic attributes were intentionally retained in the dataset, as the ensemble framework naturally distributes their influence during training, preventing instability without requiring explicit decorrelation techniques.

Model training was performed using EMBER’s ensemble of regression trees, where hyperparameters governing tree growth, ensemble size, and feature weighting are internally optimized and fixed within the Petrel-2024 implementation. This ensures computational consistency across runs when identical inputs are used. During training, well data acted as hard constraints at sampled locations, while seismic and geometric features controlled spatial extrapolation between wells. To prevent data leakage, seismic attributes were used strictly as predictors and never derived from or influenced by target variables, and spatial coordinates were included solely to represent large-scale trends without overriding well control.

Once trained, the model produced a conditional probability distribution for each grid cell. This distribution integrated information from well constraints, seismic responses, and geometric continuity, ensuring that predictions remained physically consistent across the reservoir volume. Deterministic property estimates, including mean and median values, were extracted from this conditional distribution to provide stable central tendencies for reservoir characterization.

Stochastic simulation was then performed by sampling from the conditional distributions to generate multiple equiprobable realizations. These realizations were controlled using spatial variability parameters such as interwell texture, vertical continuity, anisotropy ratios, orientation, and continuity constraints. Reproducibility of stochastic outputs was ensured by maintaining fixed random seeds for baseline realization sets, while variability was introduced only through controlled changes in spatial parameters. No recalibration or retraining of the model was performed during this stage, ensuring that uncertainty reflects geological variability rather than algorithmic instability.

Model validation and quality control were conducted using built-in Petrel-2024 diagnostics, including comparison of predicted and observed distributions, training statistics, and uncertainty envelopes defined by P10–P90 ranges. Across all properties, the QC analysis confirmed the absence of systematic bias, stable convergence behavior, and consistent reproduction of spatial trends.

**Fig. 3:**
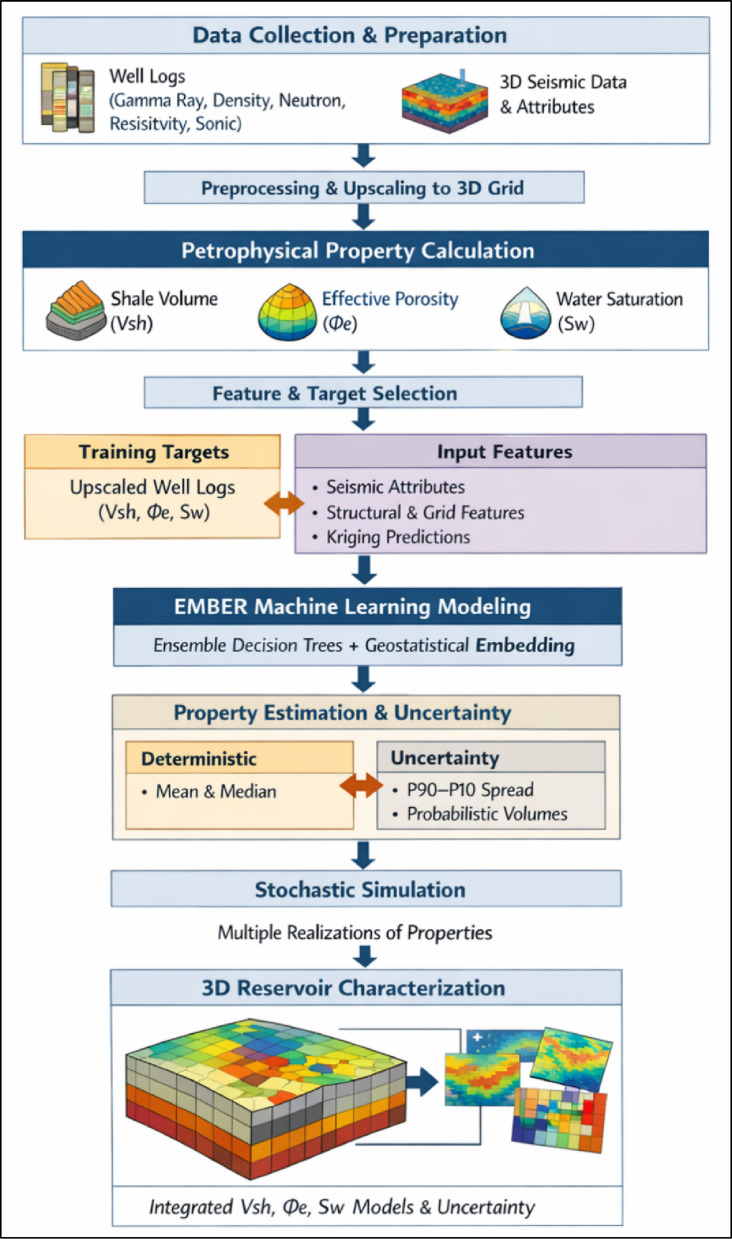
Schematic representation of the EMBER workflow illustrating the integration of ensemble machine learning and geostatistical prediction to model reservoir properties while preserving stratigraphic and spatial continuity.

Overall, the EMBER workflow constitutes a fully integrated and reproducible machine learning geostatistical framework in which seismic attributes, structural information, and well-log-derived properties are combined in a controlled ensemble learning environment. The approach inherently mitigates multicollinearity, avoids manual parameter bias, and ensures that uncertainty quantification is physically meaningful. Reproducibility is guaranteed through fixed software implementation, consistent input datasets, and deterministic workflow design, ensuring that identical inputs yield statistically consistent reservoir property models within numerical tolerance (Fig. 3).

### Model training and validation

The EMBER models were trained using the available well data through an ensemble learning approach, in which multiple decision trees are combined to produce reservoir property predictions consistent with the dataset. Model performance was assessed using internal quality control metrics generated during training, including variable importance and prediction consistency.

To evaluate predictive capability away from well control, blind well testing was implemented by excluding selected wells during training and comparing predicted properties at these locations with observed values. Machine learning–based property modeling was performed using the EMBER engine, which leverages an ensemble of tree-based estimators to capture nonlinear relationships between petrophysical properties and their controlling geological and geophysical features.

The trained EMBER models produce multiple outputs at each grid cell; however, the accuracy, particularly for water saturation (Sw), should be interpreted with caution given the limited amount of well data available for training. The blind well validation provides an initial indication of predictive performance, but additional validation with more extensive datasets is recommended to assess model robustness across the entire reservoir. The estimation mean represents the arithmetic average of predictions from all decision trees, while the estimation median denotes the central tendency of the predicted distribution. The estimation spread, defined as the difference between the P90 and P10 percentiles, provides a quantitative measure of uncertainty. These outputs form the basis for subsequent stochastic simulations and uncertainty analysis.

Stochastic simulations were generated once the training targets and features were defined. The trained models provide a conditional distribution at each grid cell, from which multiple realizations can be sampled consistently with the available data. These realizations introduce heterogeneity and variability required for constraining flow simulations across the grid or within localized zones.

Simulation variability was introduced through EMBER stochastic controls governing spatial continuity and anisotropy, with a fixed number of realizations generated using unique random seeds to ensure reproducibility. Parameters controlling spatial continuity were held constant across all property models and calibrated to honor the interpreted geological trends, promoting geologically consistent lateral and vertical continuity without introducing additional tuning during simulation. Consequently, differences between realizations reflect only conditioning to data and stochastic sampling, rather than variations in model configuration.

Each EMBER model generates a dedicated folder containing mean, median, and spread outputs consistent with the training model. For validation, a blind well test was conducted by repeating the EMBER workflow while excluding one or more wells. Both simultaneous omission of multiple wells and individual well omission are supported. In this study, the Berenice_1X well was used as the blind well, and predictions were compared against upscaled logs. It should be noted that validation is limited to a single blind well, and no cross-validation or additional testing was performed. Therefore, the results should be interpreted in the context of this limitation.

Simulation Intervention allows simulation results to be biased toward a specific property or target value while preserving statistical consistency. These options are available after initial simulations and should be applied after evaluating baseline realizations.

### Estimation and uncertainty quantification

Three metrics were used to evaluate agreement between modeled outputs and blind well data. Root Mean Square Error (RMSE) measures the square root of the average squared differences between predicted and observed values, with lower values indicating better performance. Mean Absolute Error (MAE) represents the average absolute difference between predictions and observations. The coefficient of correlation (R) quantifies the strength of the linear relationship between predicted and observed values, ranging from 0 to 1.

The EMBER workflow produces both deterministic estimates and stochastic uncertainty representations. Deterministic outputs include the estimation mean and median, while uncertainty is quantified using the estimation spread (P90–P10). In addition, probabilistic outputs such as quantiles and probability volumes were generated to further support spatial uncertainty assessment and reservoir risk analysis.

The final EMBER-derived property models and uncertainty volumes were integrated within the 3D geological framework of the Berenice Field. This workflow enables a consistent, data-driven characterization of shale volume, effective porosity, and water saturation while explicitly accounting for geological heterogeneity and uncertainty. The methodology provides a transferable approach applicable to other structurally complex reservoirs in Egypt’s Western Desert.

## Results and discussion

### Petrophysical properties of the AEB-3E reservoir

Petrophysical analysis of the AEB-3E reservoir was conducted using composite well logs from the four available wells. Key properties, including shale volume (Vsh), effective porosity (Φe), and water saturation (Sw), were derived following standard industry workflows using Gamma Ray, Neutron (NPHI), Density, and Resistivity logs. These properties were used to evaluate reservoir quality and hydrocarbon potential across the studied interval.

Shale volume was primarily calculated from gamma ray responses using the linear method, revealing generally low shale content across the AEB-3E sub-member, ranging from approximately 0.1 to 0.65. Quantitatively, Berenice–TD-1X exhibits the highest shale volume (Vsh = 0.18), followed by BERENICE-03 (0.16), while lower values are observed in BERENICE-08 (0.12) and BERENICE-09 (0.11), indicating relatively cleaner reservoir intervals in the latter wells.

Effective porosity was obtained from density–neutron relationships after shale correction, highlighting variations in reservoir quality between wells. BERENICE-08 and BERENICE-09 record the highest effective porosity values (Φe = 0.147 and 0.142, respectively), followed by Berenice–TD-1 × (0.13), while BERENICE-03 shows the lowest value (0.11).

Water saturation was estimated from resistivity measurements using the Indonesian model, calibrated to lithology and fluid properties. The calculated Sw values show clear differences between wells, with BERENICE-08 recording the highest value (Sw = 0.45), followed by BERENICE-09 (0.37) and BERENICE-03 (0.32), while Berenice–TD-1X exhibits the lowest saturation (0.28).

Vertical distributions of these properties show notable heterogeneity within the AEB-3E interval. Shale content tends to increase toward the base, while porosity and hydrocarbon saturation are more pronounced in the upper and middle units. Lateral variations among wells reflect differences in depositional facies and potential compartmentalization influenced by structural features.

**Fig. 4:**
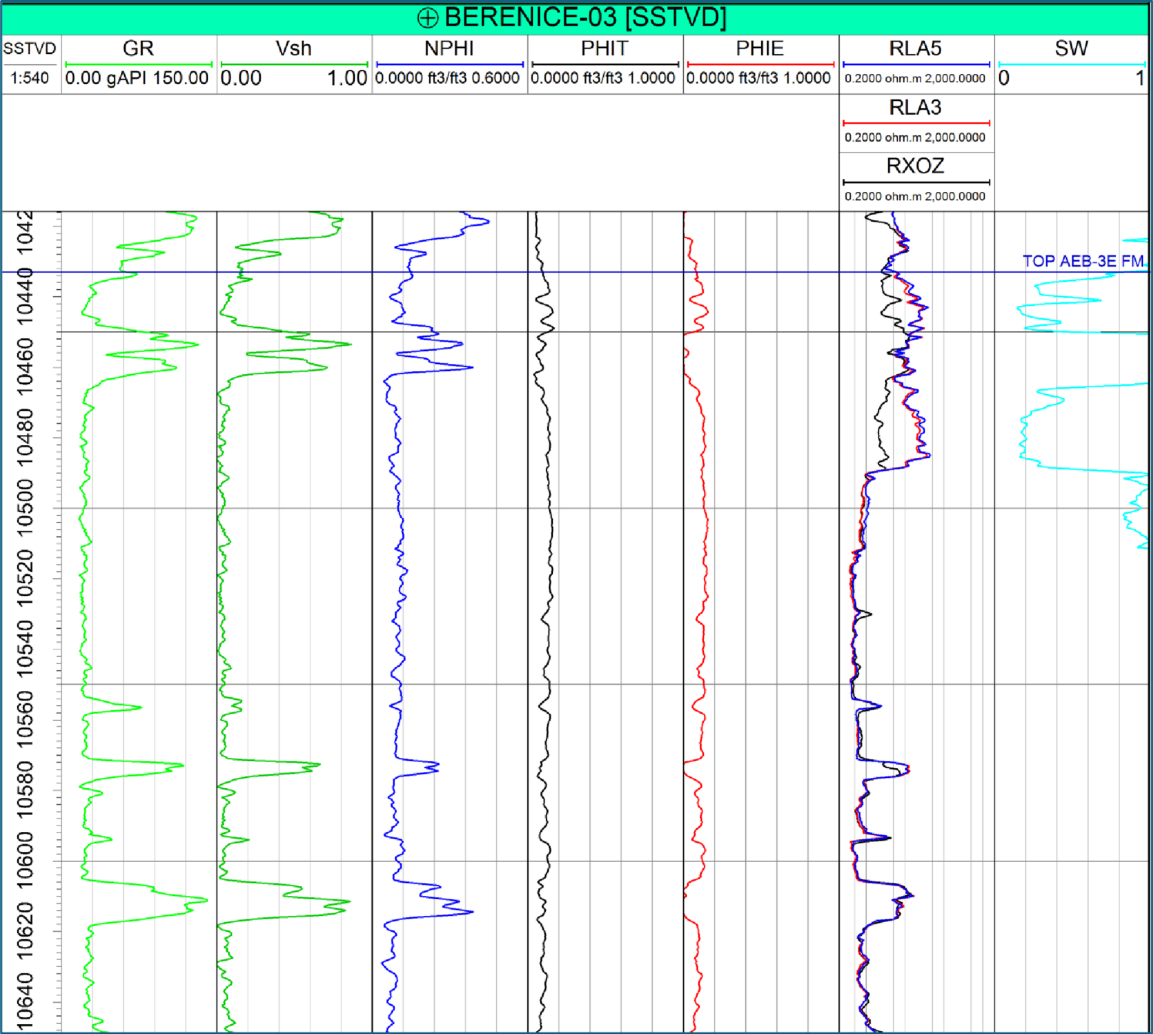
Vertical distribution of shale volume (Vsh), effective porosity (Φe), and water saturation (Sw) from the Berenice-03 well, illustrating key heterogeneity within the AEB-3E reservoir and highlighting intervals of high reservoir quality and potential hydrocarbon accumulation.

To illustrate these trends, an example from the Berenice-03 well is shown in Fig. 4, highlighting the vertical profiles of Vsh, Φe, and Sw. These properties were subsequently upscaled to the reservoir grid to serve as target variables for the machine learning workflow, ensuring that both vertical trends and lateral heterogeneity are captured in the 3D property model.

### Structural model

**Fig. 5:**
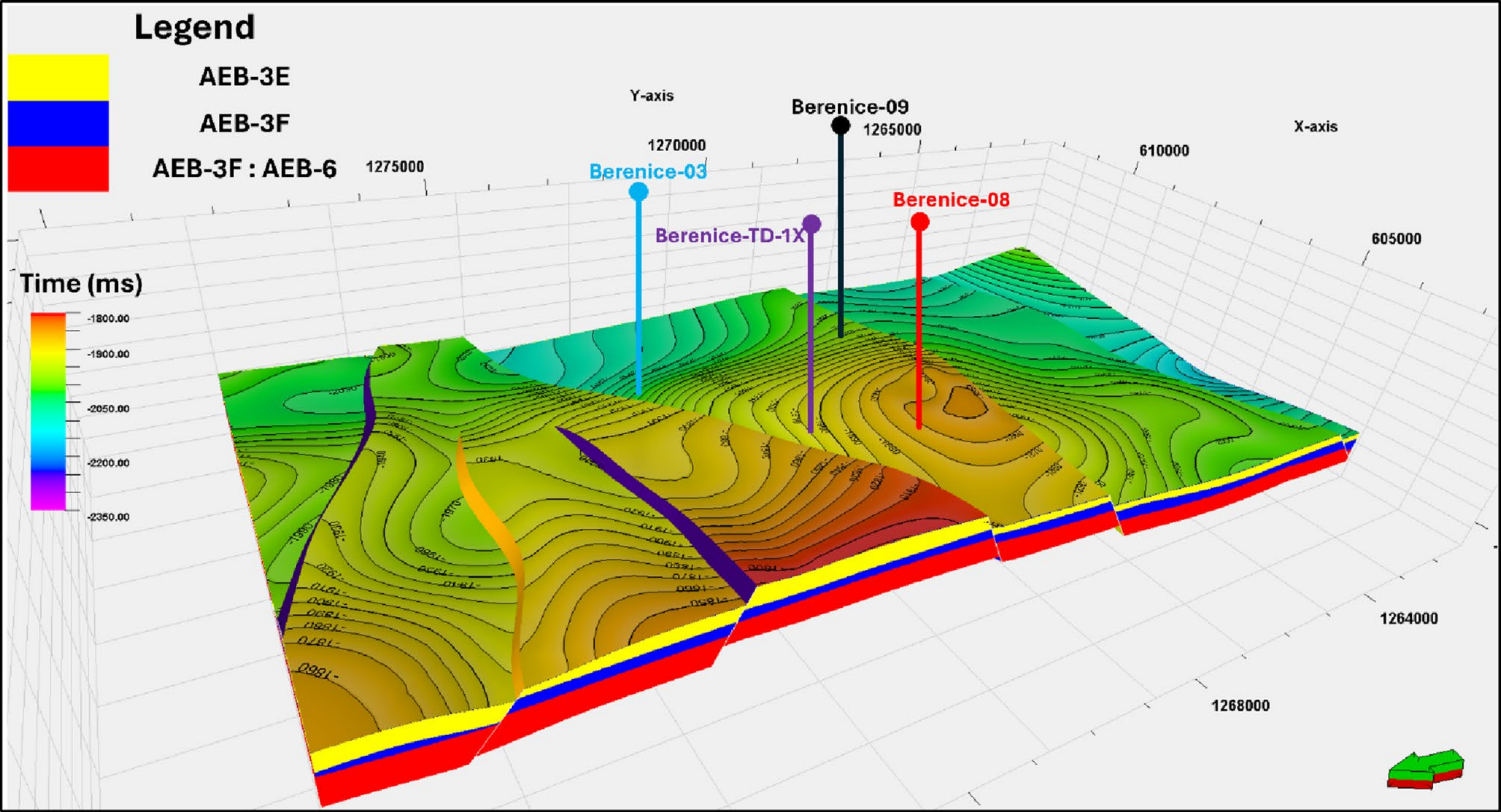
Structural framework of the Berenice Field illustrating the subdivided formations from AEB-3E to AEB-6, major fault trends, and key structural features, including half grabens and horsts, providing the geometric context for reservoir characterization.

A 3D structural framework of the Berenice Field was constructed to provide the geometric foundation for integrating well and seismic data in reservoir modeling. The model spans the interval from the top of AEB-3E to the top of AEB-6 (Fig. 5), within which the AEB-3E sub-member was extracted for property modeling.

The reservoir interval was discretized into 50 vertical layers to adequately represent stratigraphic variability and ensure consistent resolution for subsequent petrophysical modeling. Seismic-interpreted faults were incorporated to define structural boundaries and account for compartmentalization affecting lateral continuity of reservoir properties.

### EMBER model training and blind well validation

To predict the spatial distribution of key reservoir properties across the Berenice Field, the EMBER machine learning workflow was employed. Three wells were used to train the models, with the fourth well, Berenice_1X, reserved as a blind well for independent validation. This approach allowed assessment of the model’s predictive capability in areas beyond direct well control.

The training targeted three fundamental petrophysical properties: shale volume (Vsh), effective porosity (Φe), and water saturation (Sw). Upscaled well logs from the training wells served as the primary input features, while a suite of seismic-derived attributes including (variance, RMS amplitude, Chaos, Seismic-Edge, Relative Acoustic Impedance, and Second Derivative) were incorporated as secondary features. In addition, EMBER automatically integrated geometric grid properties to capture large-scale spatial trends.

The trained models generated property estimates across the entire reservoir grid, producing deterministic outputs represented by the mean and median values, along with the spread (P90–P10) to quantify predictive uncertainty. In addition, stochastic simulations were performed to capture lateral and vertical heterogeneity as well as interwell variability, which are important for reservoir characterization and subsequent flow simulation within the constraints of the available dataset. Blind well validation at the Berenice_1X well demonstrates strong agreement between the modeled and observed petrophysical properties (Table 2).

**Fig. 6:**
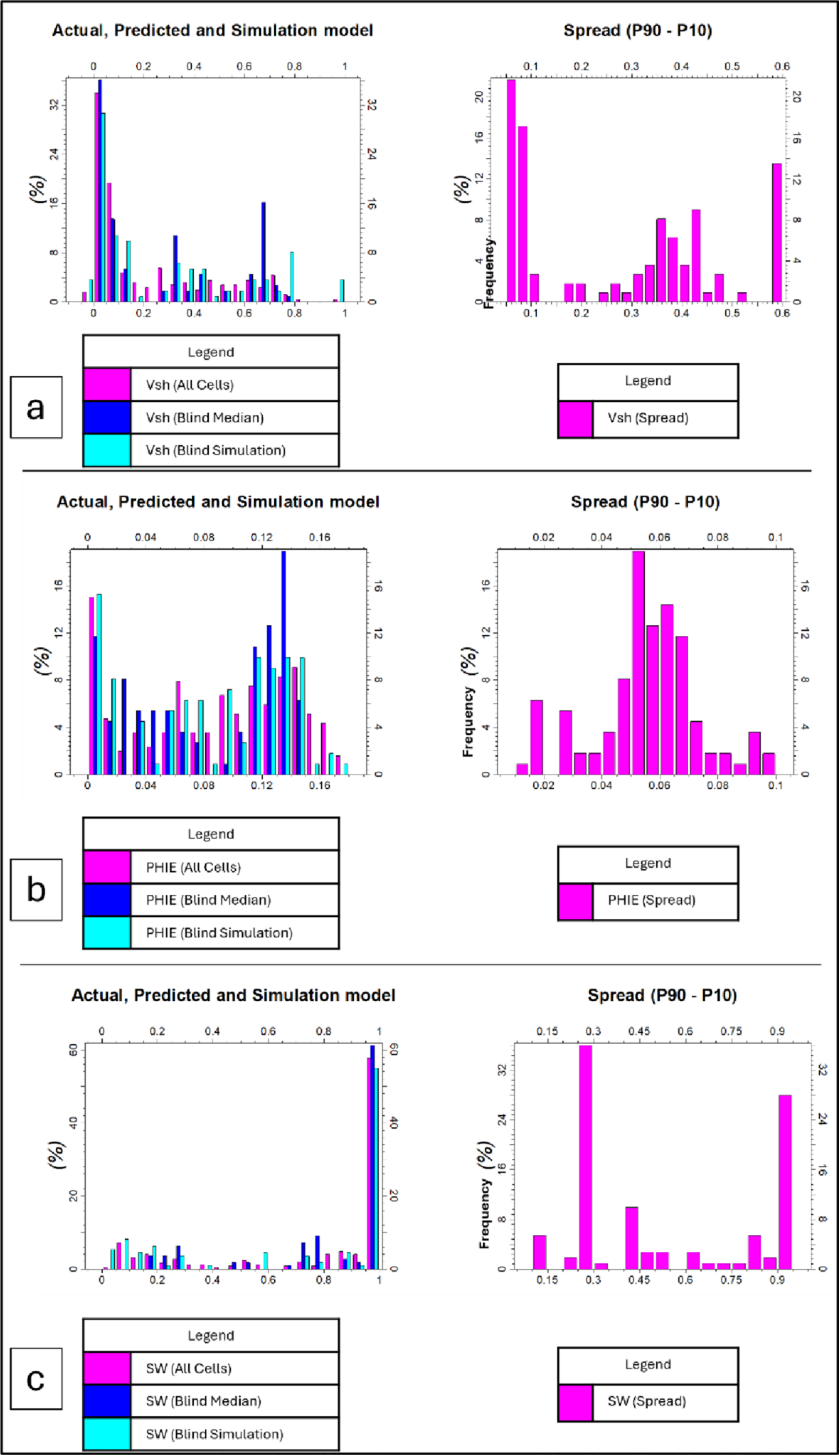
Comparison between actual (blind well data from the Berenice_1X well), predicted, and simulated petrophysical models for (**a**) shale volume (Vsh), (**b**) effective porosity (PHIE), and (**c**) water saturation (Sw). The figure also presents the corresponding uncertainty spread outputs (P90–P10) for the three parameters, illustrating spatial variability and prediction uncertainty within the reservoir.

Shale volume (Vsh) exhibits the highest correlation coefficient (*R* = 0.88), indicating reliable shale prediction, while effective porosity (PHIE) shows the lowest error values (RMSE = 0.031 and MAE = 0.022), reflecting the model’s strong performance in porosity estimation. Water saturation (Sw) predictions also display good correlation (*R* = 0.82), with relatively higher error values, which is expected given the complex and nonlinear behavior of saturation distribution. The comparison between predicted, simulated, and observed petrophysical properties at the blind well is illustrated in Fig. 6.

**Fig. 7:**
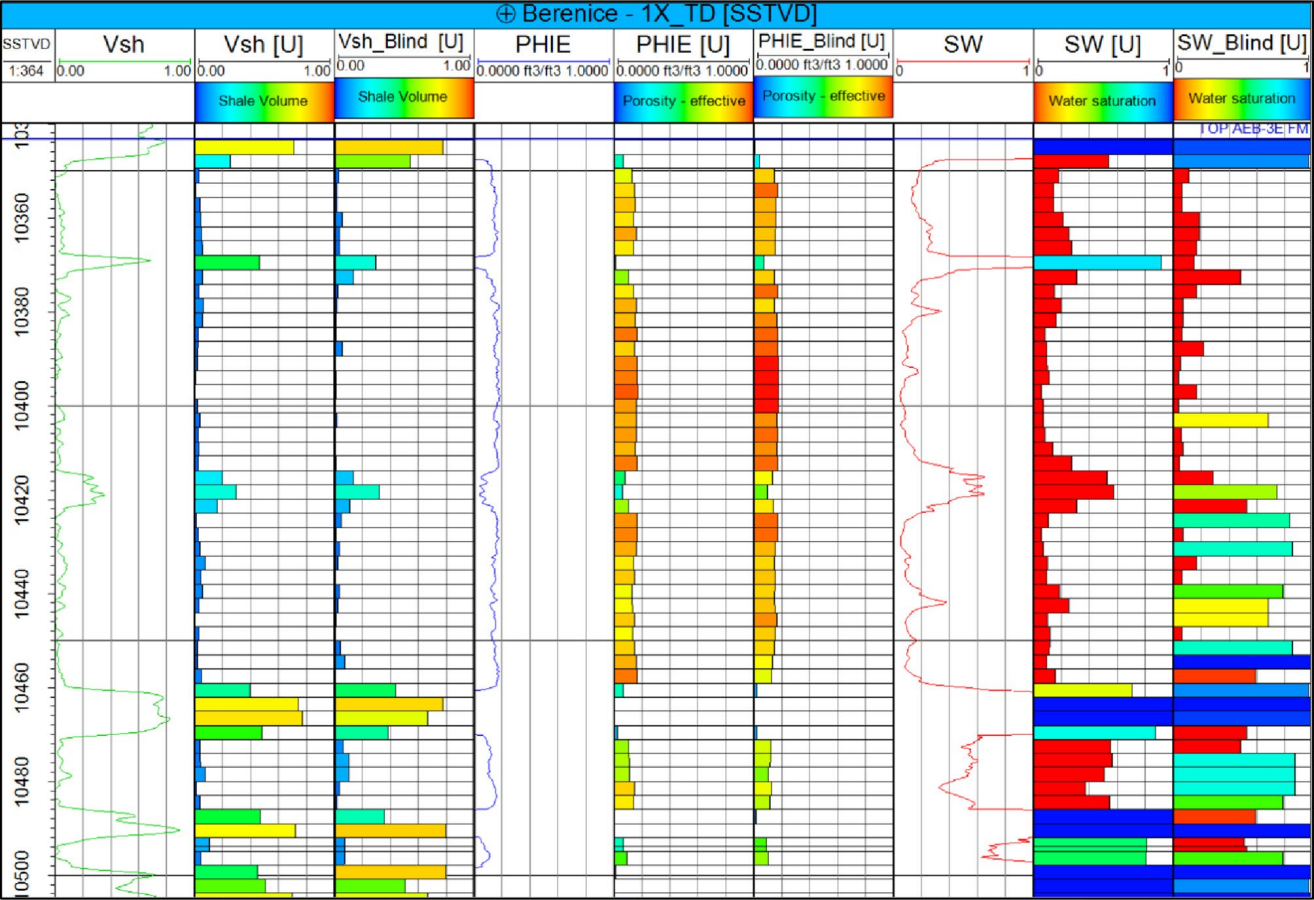
Upscaled well log responses and blind well test results for the EMBER models at the Berenice-1X well, illustrating vertical averaging of petrophysical properties from log scale to simulation grid scale and comparing predicted and observed shale volume (Vsh), effective porosity (Φe), and water saturation (Sw) away from well control.

In this study, total porosity (Φt), upscaled from the neutron porosity (NPHI) log, was incorporated as a secondary feature in both the Vsh and effective porosity (Φe) models. This integration enhanced the model’s ability to capture the shale–porosity relationship, particularly in mixed lithologies where shale content exerts a direct control on porosity distribution. Consequently, both Vsh and Φe exhibit prediction accuracies of 88% and 85%, respectively, indicating consistency within the available dataset and suggesting a reasonable performance of the selected feature set and the relationships between petrophysical parameters. In contrast, water saturation (Sw) shows a comparatively lower agreement (82%), which is expected given its weaker dependency on porosity alone and its stronger sensitivity to additional factors such as fluid distribution, resistivity response, and saturation-height effects. Unlike Vsh and Φe, Sw is influenced by complex petrophysical interactions that are not fully captured through porosity-based features, particularly when resistivity-derived uncertainties are propagated during upscaling. As a result, the reduced correlation for Sw reflects the inherent complexity of saturation modeling rather than a limitation of the EMBER workflow itself. Nevertheless, the model successfully reproduced the main vertical trends and lateral variability of Sw across the reservoir, supporting its overall predictive reliability. These results highlight the effectiveness of combining upscaled well logs and seismic attributes in an ensemble machine learning framework to produce a detailed, data-driven representation of the AEB-3E reservoir. The blind well validation suggests that the model can extend predictions into areas away from direct well control, providing a basis for uncertainty analysis and reservoir modeling within the constraints of the available dataset. (Fig. 7)

**Table 2:** Blind well validation metrics for the Berenice_1X well comparing modeled outputs with observed well data.

Parameter	RMSE	MAE	*R*
Vsh	0.147	0.088	0.88
PHIE	0.031	0.022	0.85
Sw	0.198	0.116	0.82

### Reservoir property models generated by EMBER

After training and validating the EMBER models using the three wells and confirming predictive accuracy through the blind well Berenice_1X, the workflow was applied to generate property models for shale volume (Vsh), effective porosity (Φe), and water saturation (Sw) across the AEB-3E interval. The simulation parameters described earlier were applied to ensure realistic lateral and vertical continuity, capturing both fine-scale heterogeneity and large-scale reservoir trends.

**Fig. 8:**
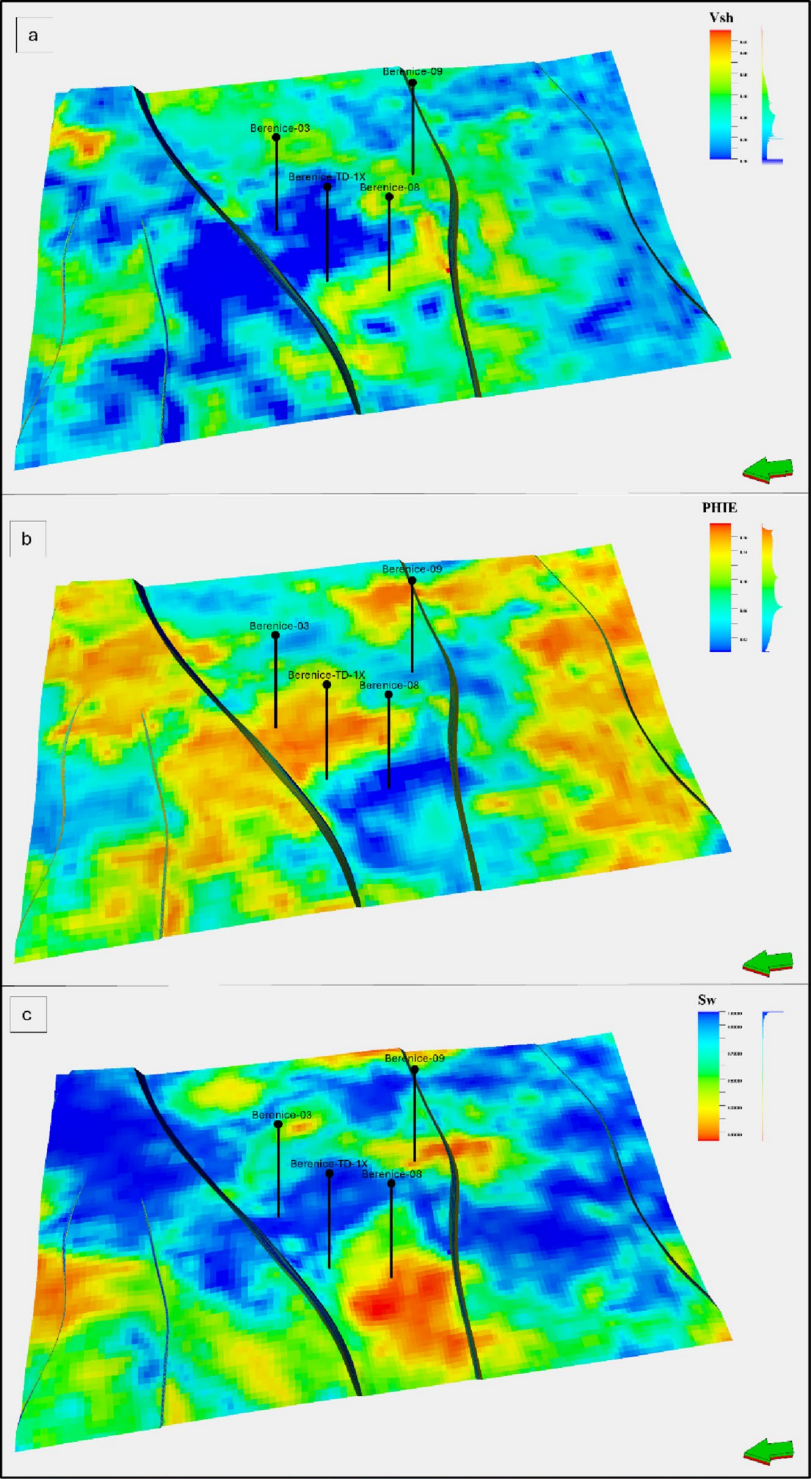
EMBER-derived property models for the AEB-3E interval in the Berenice Field: (**a**) Shale volume (Vsh) distribution highlighting low-shale zones in blue and minor high-shale patches in red, indicating predominantly clean reservoir intervals; (**b**) Effective porosity (Φe) showing fair to good storage capacity with higher values generally corresponding to low shale content; (**c**) Water saturation (Sw) illustrating lower saturation in areas of reduced shale volume and higher porosity, confirming the presence of high-quality reservoir rocks and a relatively homogeneous reservoir.

The Vsh model shows that the AEB-3E interval is largely composed of clean sands, with shale volume values ranging from 0.1 to 0.65 and most of the reservoir exhibiting intermediate values around 0.35. Zones with lower shale content (0.15–0.30) dominate the reservoir, while minor patches of higher shale (highlighted in red in Fig. 8a) are scattered throughout the field. The spatial distribution indicates a reservoir that appears to be laterally consistent within the studied wells, with minimal shale heterogeneity, suggesting favorable conditions for hydrocarbon flow. Areas of low shale content generally align with higher effective porosity and lower water saturation, consistent with expectations for high-quality reservoir zones.

The effective porosity model reveals values between 0.02 and 0.16, indicating fair to good storage capacity across the reservoir. High-porosity zones are predominantly associated with lower shale content, reinforcing the positive correlation between clean sands and reservoir quality. The overall distribution is relatively uniform, with minor variations controlled by lithology and structural trends (Fig. 8b). The consistency between Vsh and Φe demonstrates that the EMBER model effectively reproduces interwell heterogeneity, capturing both vertical layering and lateral variations by integrating seismic attributes, grid-based geometry, and structural features as training inputs.

Water saturation ranges from 0.50 to 1. Lower Sw values are primarily located in the northwest portion of the field, coinciding with areas of low shale content and higher effective porosity (Fig. 8c). This inverse relationship between Sw, Vsh, and Φe confirms the presence of high-quality reservoir rocks within the AEB-3E interval. The spatial consistency of water saturation with shale volume and porosity supports the interpretation of a largely clean and relatively homogeneous reservoir, highlighting zones favorable for hydrocarbon production.

These models suggest that the interval is predominantly clean, with indications of fair to good effective porosity; however, due to the small number of wells and the inherent uncertainties, these results should be considered preliminary. The deterministic and stochastic outputs are consistent within the current dataset, but broader validation is needed to confirm their reliability.

### Cross-sectional analysis

**Fig. 9:**
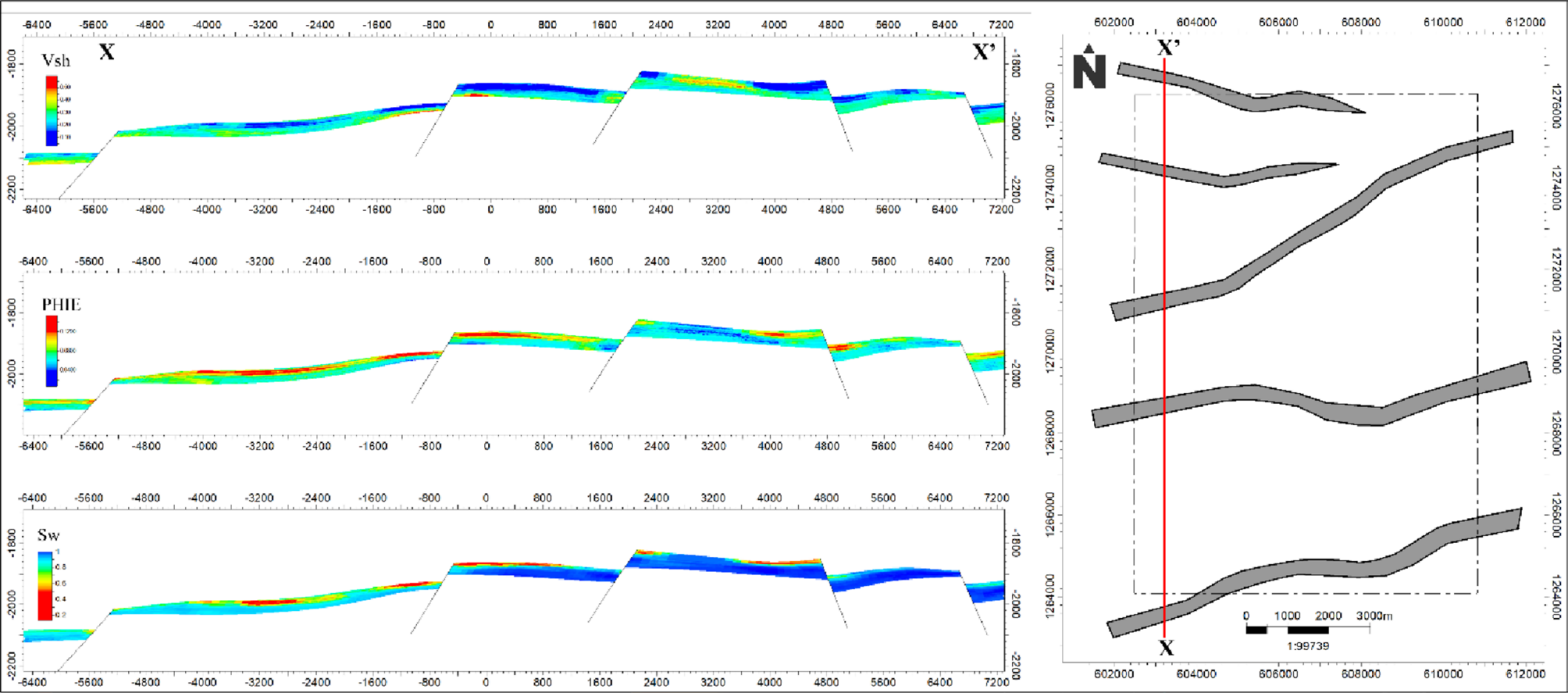
Western cross-section (X–X′) through the AEB-3E interval showing EMBER-modeled reservoir properties. Low shale volume zones correspond to higher effective porosity and lower water saturation, while structural features such as minor faults and half grabens are clearly represented, illustrating vertical and lateral heterogeneity.

**Fig. 10:**
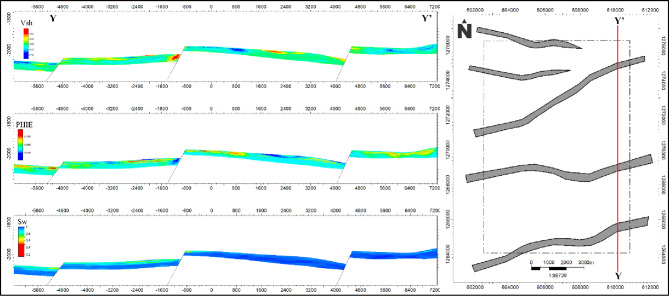
Eastern cross-section (Y–Y′) of the AEB-3E interval depicting EMBER-derived petrophysical properties. The cross-section highlights low-shale, high-porosity zones, and captures structural heterogeneity, providing insights into lateral reservoir trends and hydrocarbon-favorable areas.

To better visualize lateral variations in reservoir properties, two representative cross-sections were extracted from the EMBER models: X–X’ (western part) shown in Fig. 9 and Y–Y’ (eastern part) shown in Fig. 10. The cross-sectional analyses indicate potential lateral trends and high-quality zones, but the interpretations are provisional given the limited well control. Further data acquisition and validation are necessary to substantiate these inferred features.

The most favorable reservoir intervals are identified where these properties coincide, representing cleaner sandstone units with enhanced storage capacity and reduced water content. These intervals exhibit a reasonable degree of lateral continuity, although localized heterogeneities are present, likely influenced by structural elements such as minor faults and compartmentalization.

A clear southwestward trend of improved reservoir quality is observed across the cross-sections, characterized by the co-occurrence of lower shale content, higher porosity, and reduced water saturation. This trend defines the southwestern part of the study area as the most prospective zone for future development. Notably, this area remains undrilled, indicating a potential untapped target for hydrocarbon exploration.

Structural features observed within the cross-sections influence the distribution and continuity of reservoir properties and should be considered in future well planning to optimize reservoir penetration and connectivity.

### Impact of the EMBER machine learning workflow

The application of the EMBER workflow indicates potential advantages over conventional geostatistical approaches, particularly in reducing modeling time and capturing nonlinear property relationships. However, due to the limited well data, these benefits should be considered preliminary, and additional validation with more extensive datasets is advisable before widespread implementation.

Unlike traditional methods that rely primarily on variogram-based interpolation and require extensive manual calibration, EMBER integrates well log measurements, seismic attributes, and structural trends within an ensemble machine learning framework to generate more data-driven and adaptive property models while preserving spatial and stratigraphic continuity.

The workflow appears to facilitate efficient modeling and reduces manual effort; however, its performance in complex, data-sparse reservoirs should be validated with additional datasets to confirm its reliability and applicability beyond the current case study. In addition, EMBER captures nonlinear relationships between input variables that are often difficult to represent using traditional geostatistical methods.

The stochastic simulations produced by EMBER provide multiple realizations that account for uncertainty and interwell variability, offering a more comprehensive representation of reservoir heterogeneity compared to single deterministic outputs commonly obtained from conventional workflows. This capability supports improved data-driven decision-making and risk assessment, particularly in structurally complex reservoirs or areas with limited well control.

Overall, EMBER enhances both the efficiency and reliability of reservoir characterization, making it a practical and effective tool for detailed property modeling in the Berenice Field and similar geological settings.

### Limitations

While the EMBER-based models demonstrate reliable performance in estimating reservoir properties and capturing both lateral and vertical heterogeneity, several limitations of this study should be acknowledged. First, the primary limitation of this study branches from the small number of well controls, which restricts the spatial coverage and increases the uncertainty of the model predictions. As a result, caution should be exercised when extrapolating these results to unsampled areas. This limited number of wells restricts the size and spatial coverage of the training dataset, which may increase prediction uncertainty, particularly in areas located far from direct well control. In addition, the relatively small dataset increases the potential risk of overfitting, whereby the model may capture noise or local patterns in the training data that do not generalize well to unsampled locations. The model results are also sensitive to the selection of input features, and variations in the choice or quality of seismic attributes and other predictors may influence the stability and accuracy of the predictions.

Second, the prediction accuracy of water saturation (Sw) is comparatively lower than that of porosity- and shale-related properties. This behavior reflects the complex nature of Sw, which is less directly linked to porosity-derived features and is more sensitive to resistivity measurements, fluid distribution, and saturation-height effects. Such factors may not be fully captured through upscaled well logs and seismic-derived inputs, thereby introducing additional uncertainty into Sw predictions.

In addition, the machine learning models rely heavily on the quality and resolution of the input data, including log measurements, seismic attributes, and structural interpretations. Any uncertainties associated with log calibration, seismic attribute extraction, or data upscaling are propagated into the modeled reservoir properties. Furthermore, the trained models are field-specific, and their applicability beyond the AEB-3E reservoir should be approached with caution without retraining or validation using additional datasets.

Although the integration of seismic attributes and structural information enables interpolation of reservoir properties between wells, the inclusion of additional well data would substantially enhance model robustness, reduce uncertainty, and improve the reliability of heterogeneity characterization. Future studies incorporating a larger number of wells and, where available, core and dynamic production data would allow finer-scale reservoir property modeling and more confident identification of high-quality zones for hydrocarbon production.

### Summary of reservoir characterization

The integration of upscaled well logs, seismic attributes, and the EMBER ensemble machine learning workflow provides a detailed and reliable characterization of the AEB-3E reservoir. The models reveal that the interval is predominantly clean, with fair to good effective porosity, low shale content, and water saturation trends favorable for hydrocarbon accumulation.

Both deterministic and stochastic outputs are consistent, suggesting that the approach captures interwell property variations and provides an assessment of uncertainty within the available dataset. By combining structural, seismic, and petrophysical inputs, EMBER offers an efficient and practical workflow for reservoir modeling, particularly in structurally complex fields with limited well control.

This workflow shows promise for application in analogous geological contexts; however, further validation with additional data is recommended before widespread adoption.

## Conclusion

This study presents a preliminary integrated characterization of the AEB-3E reservoir using the EMBER workflow, which combines well log and seismic data. A structural model was developed to establish the geometric framework for the analysis, and the reservoir was subdivided into multiple units to better represent vertical and lateral heterogeneity, as well as the potential influence of fault systems on reservoir continuity.

The EMBER workflow was applied to estimate key petrophysical properties, including shale volume (Vsh), effective porosity (Φe), and water saturation (Sw), within the study interval. Results from the available wells indicate relatively low shale content, moderate to good effective porosity, and variable water saturation conditions in the sampled locations. However, because the study is based on limited well control, these observations should be considered preliminary and may not fully represent the entire reservoir. Reservoir heterogeneity and structural compartmentalization suggest that property distribution is likely to vary beyond the currently sampled areas. Deterministic models and cross-sectional analyses indicate laterally consistent trends between the analyzed wells, while stochastic simulations provide uncertainty ranges and alternative realizations of reservoir properties, particularly in interwell areas where control is sparse.

The integration of well logs, seismic interpretation, and structural modeling within the EMBER framework demonstrates its usefulness for constructing data-constrained reservoir models in structurally complex and data-limited settings. Compared with conventional deterministic approaches, the workflow offers an improved means of incorporating uncertainty and representing spatial variability.

These findings provide a preliminary basis for understanding reservoir continuity, heterogeneity, and uncertainty, which are important considerations for future reservoir evaluation and development planning. While additional wells and dynamic production data would be required for full validation, the results suggest that the EMBER workflow can serve as a practical tool for reservoir characterization in the Berenice Field and in comparable geological settings.

## Data Availability

Data are however available from authors upon reasonable and with permission of [ The Egyptian General Petroleum Cooperation].
